# Development of an infrared array sensor-integrated laser system for precision and efficacy in medical applications

**DOI:** 10.1007/s10103-025-04510-y

**Published:** 2025-06-05

**Authors:** Batuhan Dizman, Mustafa Kemal Ruhi

**Affiliations:** Institute of Biomedical Engineering, Boğaziçi University, Istanbul, Turkey

**Keywords:** Lasers, Photothermal interactions, Temperature-controlled irradiation, Temperature sensors, IR arrays

## Abstract

Photothermal treatments require precise temperature control to achieve therapeutic effects without harming surrounding tissues. However, existing systems rely on expensive components for accuracy. This study explores whether a temperature-controlled laser system can be developed using cost-effective components while maintaining precision. The system was designed and built from scratch and then tested on samples with different optical properties to identify and address its limitations. The final design ensures accuracy without requiring a computer for monitoring and control, making the technology more accessible. Our research group developed the hardware and software for a temperature-controlled laser system. The operating algorithm was then optimized using phantom and ex vivo tissue. The IR array sensor was used with its factory calibration, and its accuracy was compared to that of another commercial IR temperature measurement device. Finally, the results of the temperature-controlled ex vivo application were compared to those obtained using corresponding constant laser power values. The developed system could distinguish between agar and lamb liver and adjust the irradiation parameters accordingly. In the experiments where the samples were kept at 42.5 °C, the mean and standard deviation of the agar gel and lamb liver tissue temperatures were 42.10 ±0.37 °C and 42.92 ±0.39 °C, respectively. Subsequent experiments demonstrated that the developed system maintained the lamb liver tissue temperature at set values, with a standard deviation of less than ± 0.48 °C. In this study, a precise, customizable, and relatively inexpensive temperature-controlled laser system was developed and tested. The operation algorithm was developed based on preliminary studies, and the precision was maintained on samples with distinct optical properties. Future studies should focus on making the device more compact and testing the system in in vivo models.

**Clinical trial number** Not applicable.

**Supplementary Information:**

The online version contains supplementary material available at 10.1007/s10103-025-04510-y.

## Introduction

Medical applications leveraging photothermal interactions aim to induce specific biological effects through localized heating, including tissue necrosis, triggering an immune response and denaturation of proteins [1, 2]. Two critical parameters determining the success of photothermal treatments are the peak tissue temperature and the duration at which the peak tissue temperature is maintained [3]. For example, hyperthermia of tumors aims to induce cancer cell death by maintaining tumor tissue at around 43 °C for a specific period, which leads to protein denaturation and disruption of the cell membrane [4, 5]. Research has also shown that maintaining the tissue between 41 °C and 43 °C increases the expression of heat-shock proteins, which then interact with antigen-presenting cells, leading to the secretion of various inflammatory cytokines and the maturation of dendritic cells [6–8]. In laser tissue welding, the incised tissue is fused with no or minimal inflammation and fibrosis [9–11]. This method is particularly useful in microsurgery, where working with traditional sutures is challenging [12]. Laser tissue welding works on the principle that partially denatured collagen molecules, due to heating with a laser, reassemble during cooling [13]. For collagen denaturation to occur, it is crucial to maintain the tissue temperature between 60 °C and 70 °C for a certain period [11]. Since collagens play a significant role in skin structure and elasticity, stimulating their production by heat therapies has also been studied for cosmetic purposes, especially in the face [14].

Precise temperature control during the above-mentioned photothermal applications is crucial for success. It is equally important to avoid unwanted tissue damage, which can lead to complications, such as thermal burns and nerve palsy [15, 16]. In studies conducted by Moritz and Henriques in the mid-1900s, it was demonstrated that tissue thermal damage can be measured using the Arrhenius integral [17]. Analyzing the Arrhenius integral reveals that thermal damage has an exponential temperature dependence. For instance, in the context of laser irradiation of tissue, performing the application at a constant power causes a linear increase in tissue temperature, leading to exponential tissue damage. On the other hand, if the temperature is constant during laser irradiation, the tissue damage will increase linearly, thus being more controlled [18, 19]. Therefore, a laser whose power is controlled according to temperature feedback, that is, a temperature-controlled laser, would decrease the risk of damage and ensure the desired biological outcome.

Different research groups have attempted to develop temperature-controlled lasers for medical applications [20–27]. Typically, the challenge is to provide precise temperature control using a low-cost setup. Additionally, it is preferred that the temperature is measured without contact to avoid the risk of contamination and interference with the laser beam. For example, two studies that worked on laser tissue welding and vascular welding using infrared thermometer feedback-controlled lasers achieved temperature stability of ± 4 °C and ± 2 °C, respectively [20, 21]. A clinical trial in the same area conducted on ten patients showed that the results of bonding skin incisions using an infrared (IR) detector feedback-controlled CO_2_ laser were comparable with sutures in terms of aesthetic appearance, pain, bonding strength, and healing time [23]. However, the error in the set temperature (65 °C) was ± 5 °C. In an in vivo laser hyperthermia study, Nomura et al. used a high-cost infrared thermal camera-controlled 808 nm laser to irradiate mouse skin [24]. The thermal signal was recorded at 50 frames per second (FPS) with a spatial resolution of 384 × 288 pixels. According to the results, the device kept the mice’s skin at the target temperature with a standard deviation of 0.14 °C. Another in vivo thermal therapy study used an IR Array-equipped laparoscopic laser system to treat liver tumors in rats [26]. Harada et al. showed that thermal therapy using this system significantly decreased the tumor volume. Inspired by Harada et al., an IR Array was selected as an inexpensive and noncontact temperature measurement method in this study.

A key contribution of this study lies in its cost-effective design strategy. Rather than relying on high-end, plug-and-play components, we selected low-cost elements and developed custom software and hardware solutions to overcome their performance limitations. This approach demonstrates technical ingenuity in extracting maximum functionality from minimal resources and offers an accessible model for other researchers or developers working with constrained budgets. Additionally, our system allows the algorithm to be configured for tissues with varying thermal properties and is compatible with monitoring applications on devices such as tablets and phones. This paper describes the hallmarks of the developed system and evaluates its performance on phantoms and lamb liver tissue by comparing temperature-controlled applications over fixed-power applications (laser current was fixed to an average value calculated from the corresponding “temperature-controlled” application). Since the system was developed and optimized through innovative design and development approaches unique to our research group, detailed descriptions of the methods are occasionally provided in the Methods section, as well as in the supplementary materials of this paper.

## Materials and methods

The developed temperature-controlled laser system consists of a power unit, a diode laser, an infrared (IR) array unit, and an electronic control unit (ECU). The laser diode was purchased from Zhuhai AIKE Photonics Technology Co., Ltd. The IR array sensor (HTPA32 × 32dR2L5.0) was purchased from Heimann Sensor GMBH and utilized in its factory-calibrated state. The remaining materials, including positioning lasers, STM32F446RCT6 microcontroller, electronic components, printed circuit boards (PCBs), positioning lasers, cases, and construction materials, were purchased from local suppliers. Autodesk Eagle was used for the PCB design, whereas STM32CubeIDE was preferred for embedded software development. The lamb livers used in ex vivo studies were purchased from a local butcher.

### Selection of the laser diode and temperature unit

Near-infrared lasers are commonly used in photothermal applications due to their effective tissue penetration and moderate absorption by water and hemoglobin. Therefore, an 808 nm continuous-wave C-Mount laser diode is preferred in this study. We aimed to select a contactless yet precise method for temperature detection. For this reason, we chose an IR array, which offers easy integration through an I2C interface. Compared to thermal cameras, IR arrays are affordable, require less processing power, and are simpler to implement. The IR array sensor used in this study was kept at its factory settings. The sensor has a resolution of 32 × 32 pixels. According to the manufacturer, the sensor has a field of view of approximately 33°, and the frame rate is adjustable between 2 and 27 Hz. The sensor was operated at 3 Hz during the experiments.

### Design of the developed temperature-controlled laser system

**Fig. 1:**
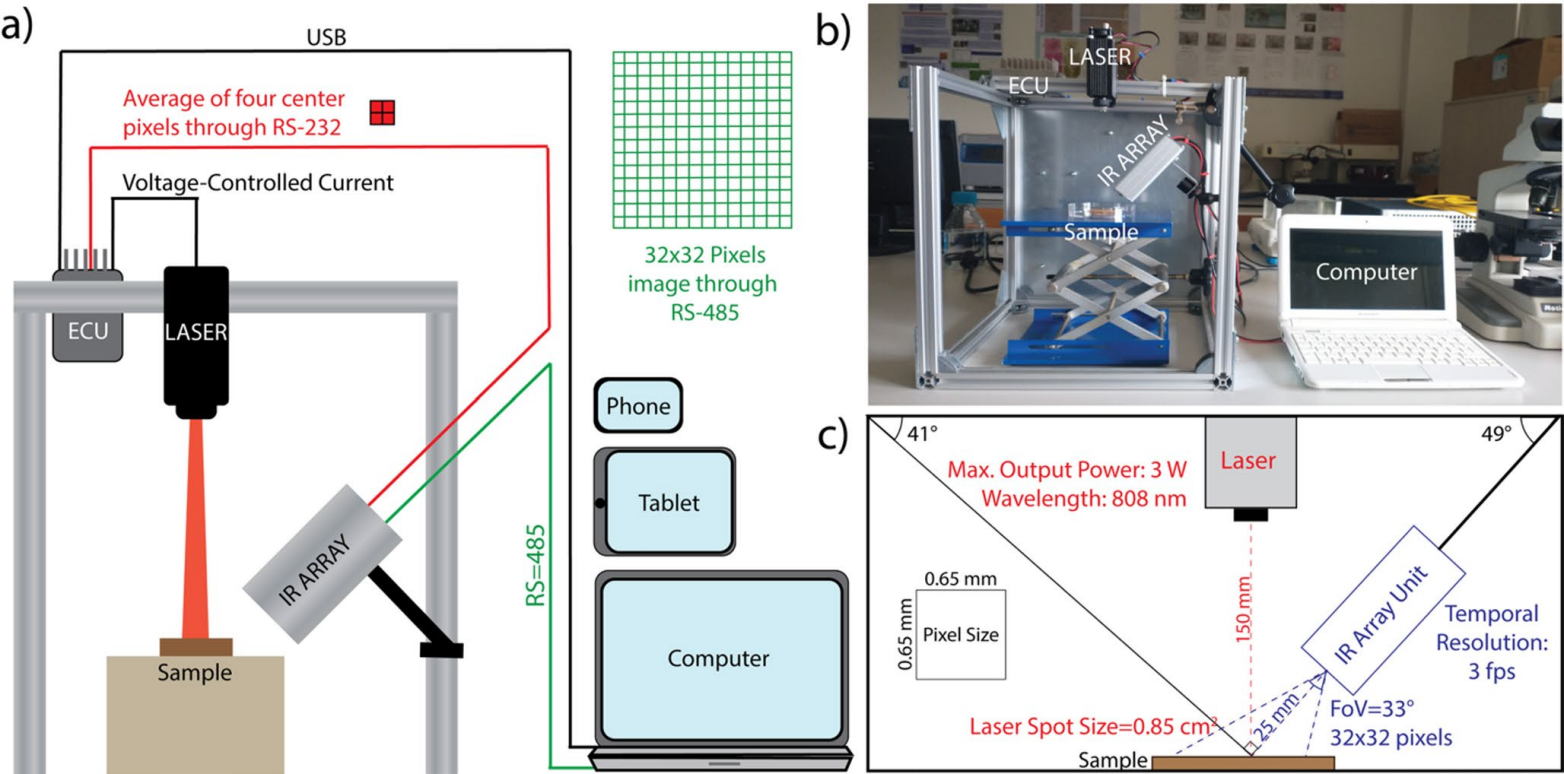
A schematic (**a**) and a photograph (**b**) of the developed temperature-controlled laser system. The electronic control unit (ECU) controls the laser via voltage-controlled current. The ECU can be connected to a personal computer (PC) or any device that operates with Linux, Windows, or Mac OS X via USB. The IR Array sensor provides both an average of four center pixels to the ECU via RS-232 or a 32 × 32 pixel image to the PC via RS-485. (**c**) Key distances and numbers about the setup. The IR array sensor was positioned 25 mm from the sample at an angle of 49° relative to the horizontal. The 808 nm diode laser was positioned 150 mm from the sample. The spot size was approximately 0.85 cm^2^

The laser was connected to the ECU designed by our research group (Supplementary Fig. 1–3), and its operation was controlled via a voltage-controlled current. The ECU has a USB interface that supports LINUX, MAC OS X, and Windows. Therefore, the device can be connected to a personal computer (PC) or mobile device via a USB for monitoring and giving commands. An IR Array unit, consisting of a PCB board, IR Array, and positioning lasers (details in Supplementary Figs. 4–6), was connected to the ECU and monitoring device, as shown in Fig. 1. The IR array unit was also connected to the monitoring device to acquire 32 × 32 pixels of the temperature data at any time. Finally, two 5 mW, 650 nm positioning lasers were mounted on either side of the IR array module to help align the laser beam with the sample.

### Preparation of the samples

This study used two samples with distinct optical properties as models. Agar gel has a lower absorption at 800 nm, with an absorption coefficient of less than 0.20 cm^− 1^, compared to liver tissue, which has 0.99 cm^− 1^ for rat and 0.80 cm^− 1^ for porcine absorption coefficient at 808 nm [28, 29].

Agar gel was prepared by dissolving 40 g of agar powder in 1 L of distilled water and poured into 100 mm Petri dishes to obtain gels with a thickness of approximately 5 mm. Fresh lamb livers were obtained from a local butcher and samples with thicknesses of 6–7 mm were prepared before the experiments. The samples were stored in saline throughout the experiments. At the beginning of each experiment, a 32 × 32 pixel thermal image acquired from the IR array sensor was used to adjust the position of the thermal camera such that the four central pixels of the laser spot corresponded to the region of highest temperature. This ensured that the temperature readings reflected the peak heating zone to avoid micro-burns while achieving tissue heating. The samples were then irradiated using a temperature-controlled laser. The maximum laser output power used in this study was 3 W, which could be considered at the upper end in some laser hyperthermia studies [30–32]. However, it should be noted that the average laser output power was lower than 3 W during the applications, particularly in ex vivo experiments.

### Preliminary experiments

First, the measurements obtained from the IR array sensor were compared with those from a commercial handheld IR temperature device (Raytek Raynger). The average temperature values measured by the two devices were found to be comparable, with a maximum difference of 2.38 ±0.61 °C when measuring the temperature of boiling water. Subsequently, an algorithm was developed to heat the agar gel and liver samples to a target temperature and maintain them at that level. This was successfully achieved but required two separate algorithms for the two sample types. This part of the study was published in a conference proceedings volume, which is listed in the references [33].

### Improvement and evaluation of the system

Preliminary experiments using agar gel and lamb liver showed that the rate of temperature increase can be drastically different depending on the optical properties of the samples. If the algorithm adjusts the power based on the algorithm designed for agar gel, the temperature of the lamb liver would increase quickly, and the tissue would burn. On the other hand, if the algorithm was adjusted for tissue in which its optical properties allow better photothermal interaction, such as lamb liver, a sample like agar gel would never be heated up to 42.5 °C mainly because the frames per second (FPS) of our IR Array unit was not fast enough to detect the surface temperature and generate an outcome.

**Fig. 2:**
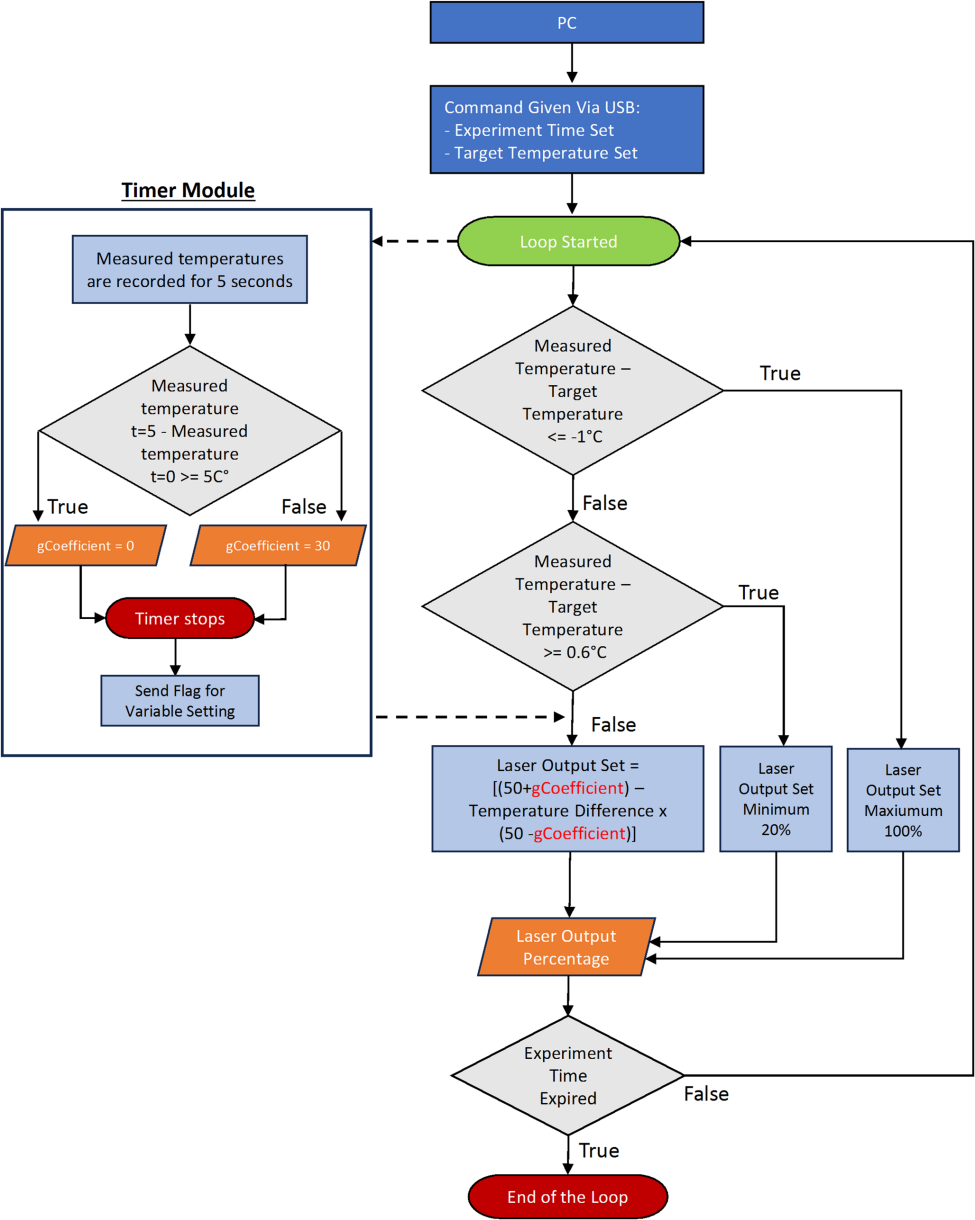
The algorithm that can distinguish between agar and lamb liver and adjust the irradiation parameters accordingly

To overcome this issue, instead of purchasing a more expensive temperature unit, we decided to benefit from the design flexibility. Because the slope of the temperature increase can be determined in the first five seconds of the experiment, an algorithm that detects the rate of temperature increase at the beginning of irradiation and decides on the next steps based on that observation would work on both samples. Therefore, the two algorithms proposed in our preliminary study [33] were combined to make a new algorithm (Fig. 2 and Supplementary Fig. 7) that detects the temperature increase rate and runs a function in the system timer module of the microcontroller, which operates simultaneously with the main program and controls the laser power.

Here, the function remains idle until a command is received from the PC. Upon receiving the command, the loop is initiated, and the temperature increase rate detection function in the timer module of the microcontroller is activated. At “t = 0,” where t represents seconds, the first temperature measurement is recorded, and at “t = 5,” the last measurement is recorded. The gCoefficient variable was then set based on the difference between these two values. If the difference is higher than 5 °C, gCoefficient remains the same, that is, zero; otherwise, gCoefficient equals 30. In other words, the gCoefficient variable irradiation algorithm was selected. This function remained idle until another command was provided.

Meanwhile, in the main function, the loop refreshes approximately every 350 ms, continuously receiving and comparing the temperature data from the IR array sensor until the duration of the experiment is complete. During the loop, the measured sample temperature (average of four pixels located at the center of the 32 × 32 pixels data) was compared with the target temperature. If the difference between the measured temperature and target temperature was smaller than − 1.0 °C (i.e., the sample temperature < 41.5 °C), the current, and therefore the laser output, was set to 100%, which refers to 3 W. If the difference between the measured temperature and the target temperature was larger than 0.6 °C (i.e., the sample temperature > 43.1 °C), the current was set to a lower value so that the output power decreased (the conversion between current and laser output power was calculated using the curve shown in Supplementary Fig. 8). For temperatures outside the specified conditions, the following formula was applied: Laser Output Set = [(50 + gCoefficient)– (Temperature Difference × (50 − gCoefficient))]. Initially, the “gCoefficient” variable was set to zero, but was adjusted at the 5th second of the application according to the optical property.

In the first irradiations, the system was tested at different target temperatures; in the second part, it was maintained at a fixed output power. In fixed-power applications, the electric current values required to achieve specific output powers were estimated by integrating the current-versus-time curves from the corresponding temperature-controlled experiments and dividing the result by the irradiation duration. The experiments were repeated three times, and new samples were used for every replicate.

## Results

The results of the temperature measurement comparison between the purchased IR array sensor and a commercial IR thermometer on ice, water at room temperature, and boiling water showed that the measurements were comparable [33].

**Fig. 3:**
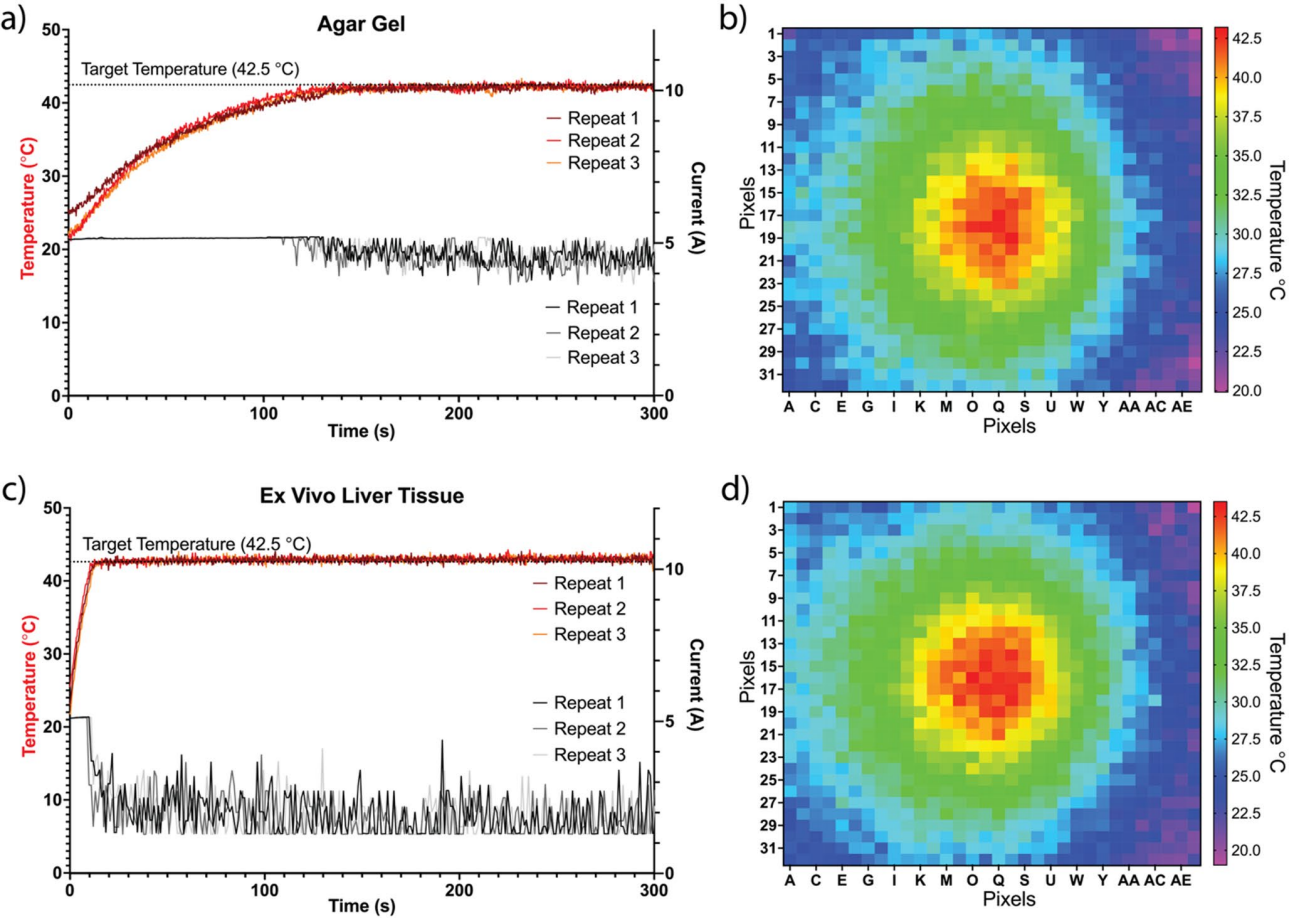
The results of temperature-controlled irradiation of (**a**) agar gel and (**c**) lamb liver. The target temperature in the algorithm was set to 42.5 °C to assess the performance of the system. The change in sample temperatures over time is shown on the left y-axis. The change in current over time is shown on the right y-axis. Each curve in the graphs represents one individual repeat. The heatmaps taken during (**b**) agar gel and (**d**) lamb liver irradiation show that the IR array covers the spots and assists in aligning the center of the image to the spot

Figure 3 shows that the algorithm, which was updated for samples with different optical properties, was able to make the selection correctly between two samples, agar and lamb liver, and adjust the power accordingly to maintain a constant temperature of 42.5 °C. The mean and standard deviation of the agar gel and lamb liver tissue temperatures were 42.10 ±0.37 °C and 42.92 ±0.39 °C, respectively. During the ECU process, debugging was performed to monitor variables, and the value of the gCoefficient, which determines the output power selection in the algorithm, was verified throughout the process.

**Fig. 4:**
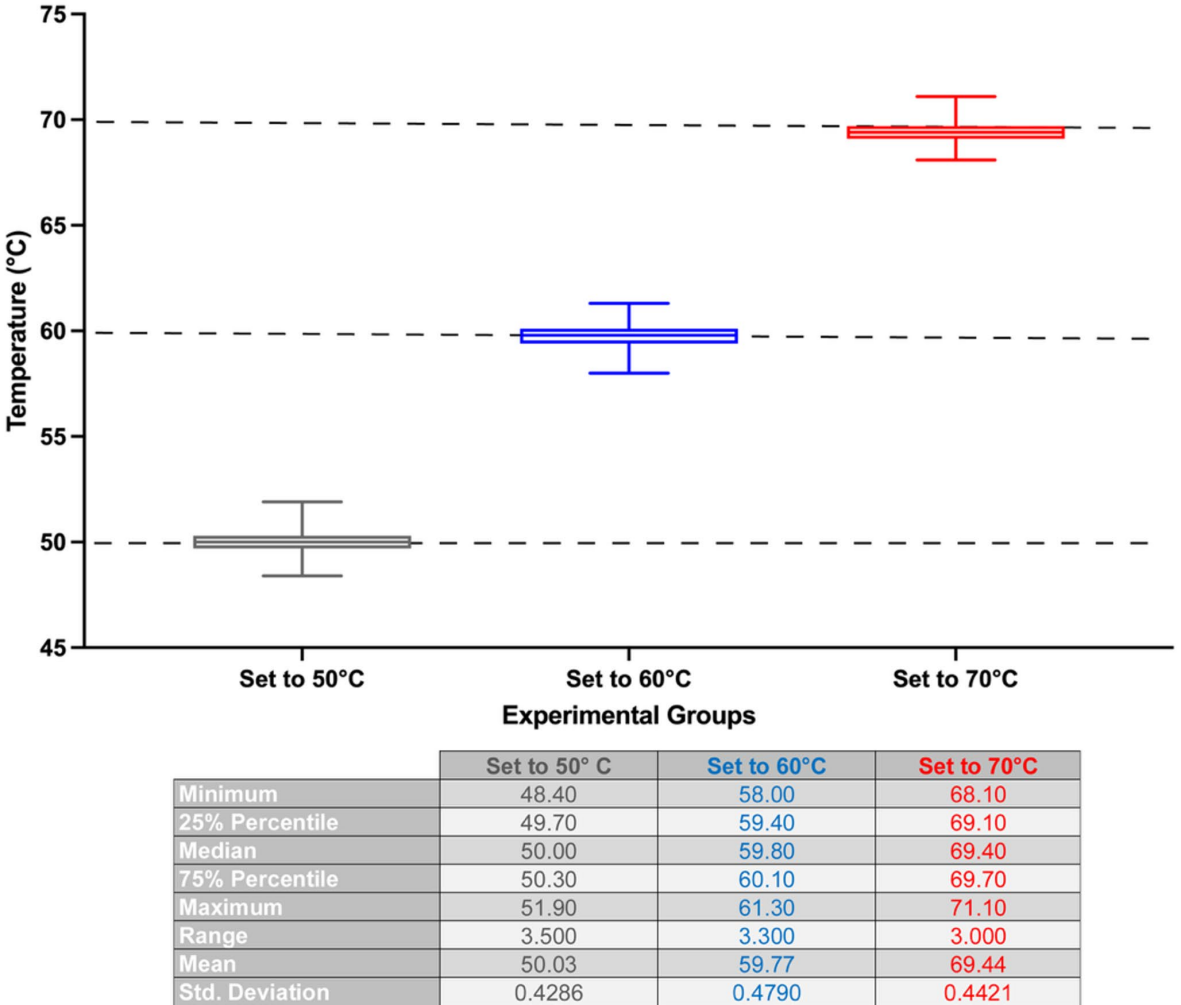
The box and whiskers representation of the results of temperature-controlled irradiation of lamb liver. The target temperature in the algorithm was set to 50 °C, 60 °C, and 70 °C to assess the performance of the system on ex vivo liver tissue. The results of three independent repeats were combined for each temperature value (50 °C, represented as gray; 60 °C, represented as blue; 70 °C, represented as red). The table underneath the graph shows the analysis details

Since the absorption of the agar at 808 nm was low, increasing the temperature of this sample to higher than 45 °C was not practical. Therefore, experiments in which the target temperatures were 50 °C, 60 °C, and 70 °C were only conducted using lamb liver. The results shown in Fig. 4 reveal that our device and the embedded algorithm successfully controlled the temperature of the lamb liver sample.

**Fig. 5:**
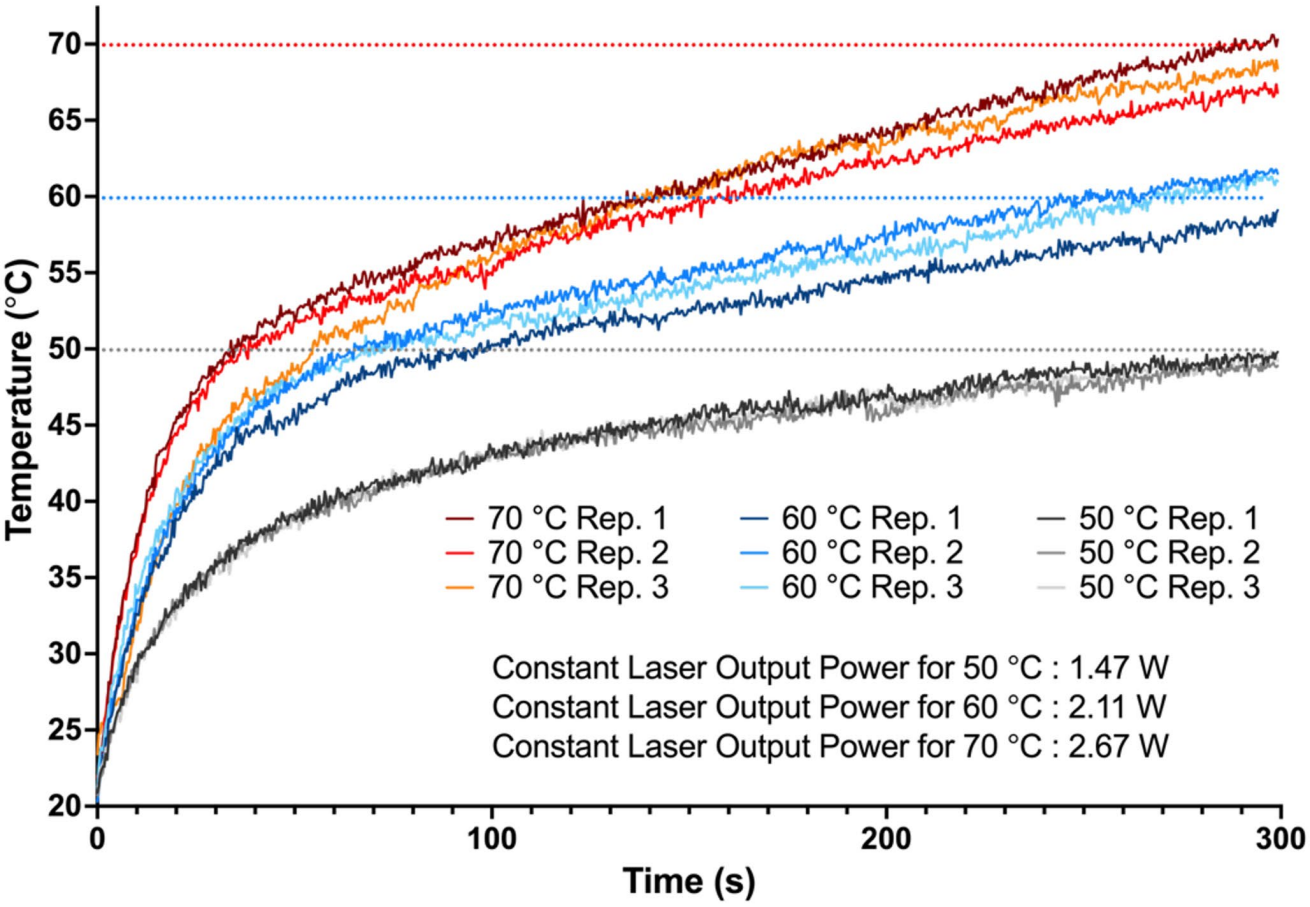
Results of the fixed-power experiment on lamb liver. Laser output power was set to 1.47 W (for 50 °C target temperature, represented as gray), 2.11 W (for 60 °C target temperature, represented as blue), and 2.67 W (for 70 °C target temperature, represented as red) for 300 s to compare the performance of the constant-power application with temperature-controlled application. To determine the electric current values required to achieve the specified constant laser output powers, the current-versus-time curves from the corresponding temperature-controlled experiments were integrated and then divided by the irradiation duration. Each curve in the graph represents one individual repeat

In the experiments that used fixed output power, however, a linear increase in temperature was observed instead of maintaining a constant sample temperature (Fig. 5).

## Discussion

Maintaining a constant tissue temperature is essential for the success of photothermal applications, such as laser hyperthermia and laser tissue welding, and for minimizing the risk of complications. To address this challenge, we aimed to develop an inexpensive and customizable temperature-controlled medical laser system. We designed a device that operates via a low-cost microprocessor (approximately $4), enabling full control of the system and compatibility with any USB-connected device, such as phones or tablets, for monitoring and command input through serial terminal software. A key objective was also to implement a precise yet affordable temperature-sensing solution. While thermal cameras with data interfaces are often expensive, IR array sensors offer a cost-effective alternative, providing accurate temperature mapping of the irradiated region. Therefore, we integrated an IR array unit that allows users to define the center of the laser spot and monitor temperature changes in its vicinity. The IR array module was acquired for approximately $100, and the laser system cost around $150. In contrast, thermal cameras equipped with data interface capabilities typically start at $500 and can exceed $2,000 for higher-resolution models. Diode laser systems vary widely, ranging from about $2,000 for entry-level units to over $3,000 for more advanced models. Considering these numbers, our system, with a total cost of approximately $500, offers a cost-effective solution, even as a single prototype.

The temperature stability of our system was ± 0.39 °C in the ex vivo experiment with a 42.5 °C target temperature. Using a thermal camera, Nomura et al. provided a better temperature stability, ± 0.1 °C, on mice skin at the same target temperature [24]. Simhon et al. aimed to keep the tissue temperature constant at 65 °C, and they achieved a temperature stability of ± 5.0 °C in their clinical study [23]. In another study, Stewart et al. investigated their laser-assisted welding system on rat aorta and achieved ± 2.0 °C temperature stability between 70 and 90 °C [21]. It should be noted that technical differences and variations in experimental parameters between studies do not allow meaningful comparisons between our results and those in the literature. Furthermore, our experimental models (agar gel and liver tissue) were only adequate for the first round of optimization of our system and should be evaluated on in vivo models, as the above-mentioned studies did.

The results indicated that the final algorithm was able to accurately distinguish between samples with different optical properties, agar, and lamb liver, and adjust the power to maintain the determined temperatures. The results of the fixed-power experiments showed that irradiation at a constant power caused a linear increase in the sample temperature, and the average temperature values were substantially lower than the target temperature, which underlines that constant power application is not ideal for laser hyperthermia and laser tissue welding, and temperature-controlled irradiation is crucial.

Although using an IR array sensor in this study enabled real-time, non-contact surface temperature monitoring, it comes with certain limitations. For example, IR temperature measurement systems are susceptible to emissivity variation, ambient light interference, dust, and surface reflections [34, 35]. Another limitation of IR-based systems is that the system is restricted to monitoring surface temperatures. Specifically, in our case, since 808 nm light can penetrate at least a few millimeters into tissue, the temperature in deeper regions may rise more than at the surface, an effect that cannot be detected by our current system [36]. There are alternative techniques that were explored in previous studies to monitor tissue temperature during laser irradiation. For example, Cipolato et al. developed a soldering material containing nanoparticles that can be used as fluorescent nanothermometers for temperature monitoring and control in effective tissue soldering [37]. Terahertz imaging has been investigated for monitoring photothermal damage during nanoparticle-assisted laser tissue soldering by Dong et al. [38]. The authors showed that a terahertz time-domain spectroscopy system could generate 3D images of the porcine skin during gold nanorod-embedded solder gel-assisted tissue soldering. Mid-infrared (MIR) techniques, microwave radiometry and magnetic resonance (MR) thermometry can also be considered as alternatives in monitoring tissue temperature [39–41]. The incorporation of these techniques could be explored in future studies to enhance the depth and precision of temperature measurement.

Compared to conventional thermal cameras, the IR array sensor used in this study has low spatial resolution (32 × 32 pixels) and lacks thermal-visual overlay capabilities, which can limit spatial interpretation in complex settings. To mitigate these issues, the sensor was placed close to the sample, and all samples were fixed in position to ensure consistent measurements. The control method used in this system also had advantages and limitations. Here, we used a proportional controller (P-controller) due to its simplicity, fast response, and low computational cost. While a PID (Proportional–Integral–Derivative) controller could offer higher accuracy and eliminate steady-state errors [42], the P-controller was sufficient for our application and easier to implement. To increase the accuracy and decrease the temperature fluctuation, PID controller can be implemented in the future. Another limitation of this study is that the thermal sensor was used with its factory calibration and was not validated against a high-accuracy thermal camera, particularly on non-uniform samples. We compared the measurements of the sensor to those of a commercial handheld device as a preliminary reference; however, a formal evaluation of accuracy, precision, and metrological performance using standardized methods is planned for future work. Additionally, this study evaluated the system’s accuracy only using agar gel and lamb liver; therefore, the impact of sample variability on accuracy warrants further investigation. Finally, it is important to note that living tissue exhibits physiological characteristics, such as active blood flow, metabolic activity, and thermoregulation, that significantly influence heat distribution and dissipation. These factors are absent in ex vivo or non-viable tissue models, which represents a key limitation of this study and will be investigated in future in vivo studies.

Overall, the current system bridges the lab-to-clinic gap primarily through its low cost. Its real-time temperature control ensures safe and precise energy delivery, which is critical for clinical procedures like laser hyperthermia and tissue soldering. Moreover, by eliminating the need for complex infrastructure or high-end components, the system supports translational research and facilitates potential clinical integration, particularly in outpatient or field settings. Features that would further support clinical translation, such as a compact design, user-friendly interface, and deep-tissue temperature measurement, are the focus of future studies.

## Conclusion

Laser-induced hyperthermia is the basis of medical applications, including hyperthermia-mediated cancer treatment and laser tissue welding. Temperature control in these applications is important to achieve the desired outcome and protect other tissues. The significance of the temperature-controlled laser system developed by our research group is that it provides precision with a relatively inexpensive setup. The primary reason for this is that the system, developed from scratch, allowed us to interfere with and customize the operation. For example, the FPS of our temperature unit was low; however, we could compensate for this by reconfiguring the algorithm that the ECU follows. In addition, our system does not require a computer to run, and the operation can be monitored through any device that has a screen and uses operating systems, including Windows and Mac OS X, via any serial terminal software. In the future, we aim to develop this system by adding interfaces such as Bluetooth connections, making the design more compact and smaller, and testing the system in in vivo studies and clinical trials.

## Electronic supplementary material

Below is the link to the electronic supplementary material.

Supplementary Material 1

## Data Availability

The data that support the findings of this study are openly available in GitHub at https://github.com/batuhankemal/Development-of-an-infrared-array-sensor-integrated-laser-system.
