# Supplementary material for: Development of an infrared array sensor-integrated laser system for precision and efficacy in medical applications
Source: Lasers Med Sci. 2025 Jun 5;40(1):254. doi: 10.1007/s10103-025-04510-y (PMC12137500; doi:10.1007/s10103-025-04510-y)
Supplement: Supplementary file 1 — Supplementary Material 1 [file 10103_2025_4510_MOESM1_ESM.pdf]

SUPPLEMENTARY MATERIALS

Supplementary Figure 1: Electronic control unit PCB design.

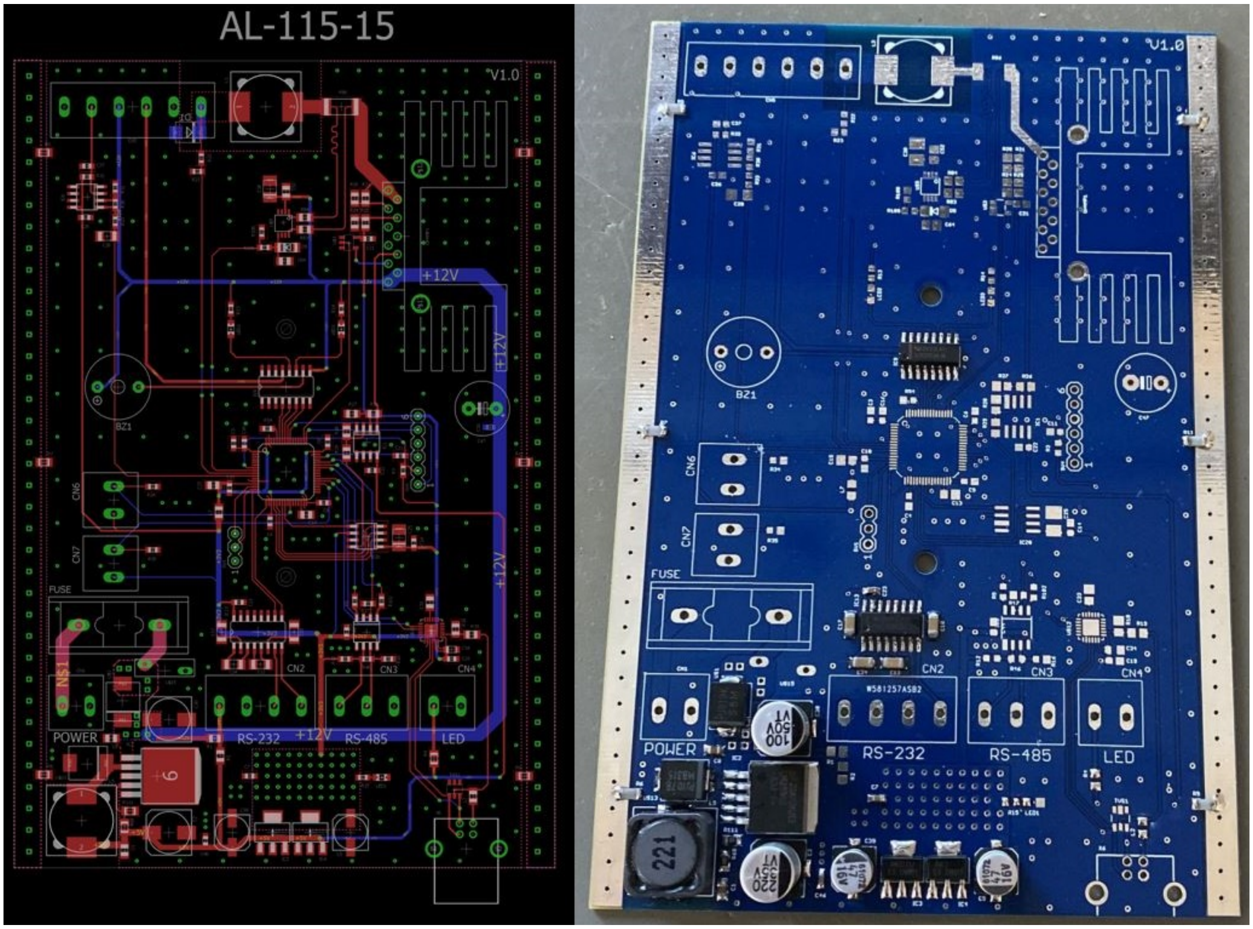

Supplementary Figure 1: Two-layer PCB design was preferred as an inexpensive solution. The PCB material was chosen as FR4 for higher temperature durability and the enclosure material was chosen as aluminum for heat dissipation. AL-115-15 code written on top of the figure shows the manufacturer product number of the enclosure. Blue lines indicate bottom layer and red lines indicate top layer. Connectors CN2, CN3 and X6 are communication interfaces. The connector CN1 is the power input of the device and the input voltage of the device is 12V. CN5 is the connector for the laser fan, laser diode and PT-500. Remaining connectors on the PCB were reserved for future use.

The PCB properties:

- Material: FR-4 TG150
- Layer: 2 Layers

- Finished copper: 35um
- Thickness: 1.6mm
- Surface finish: HASL with lead
- Size: 100.4 x 149.8 mm

Supplementary Figure 2: Electronic control unit schematics

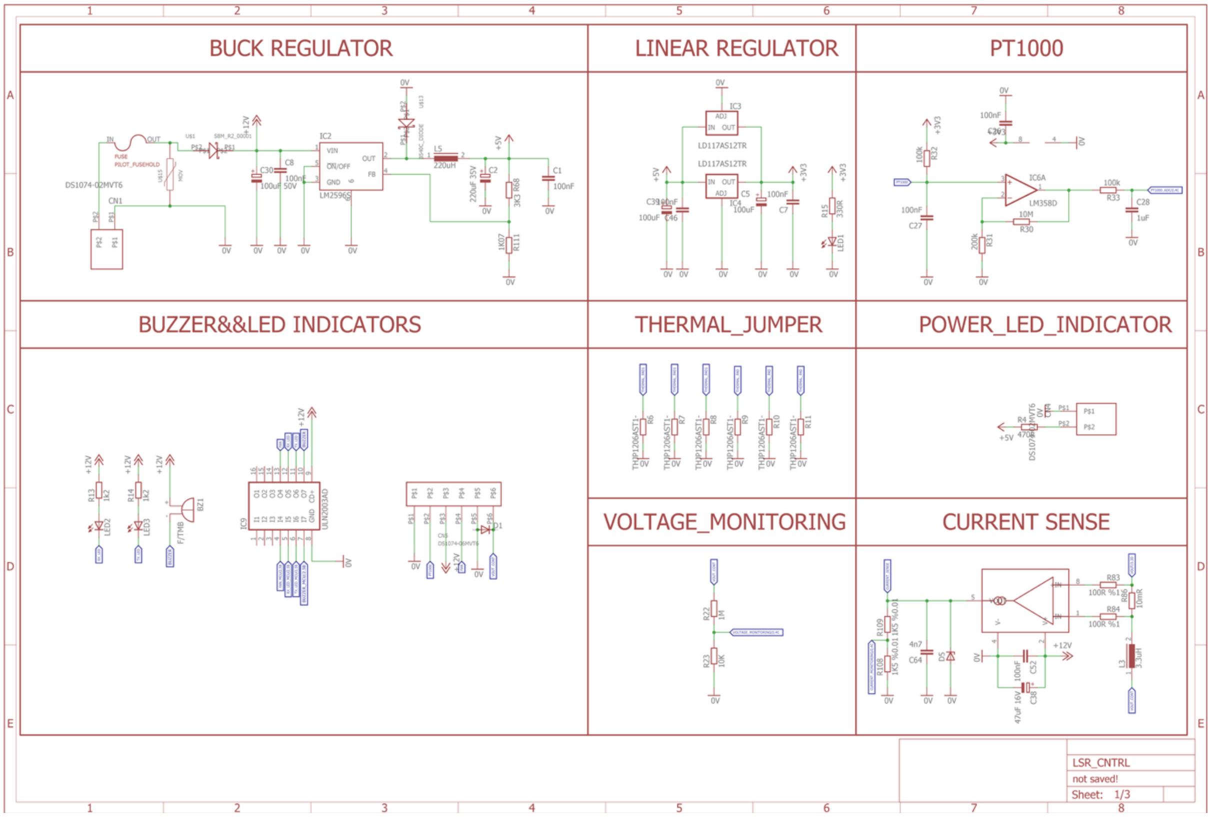

a) Electronic control unit schematics sheet1

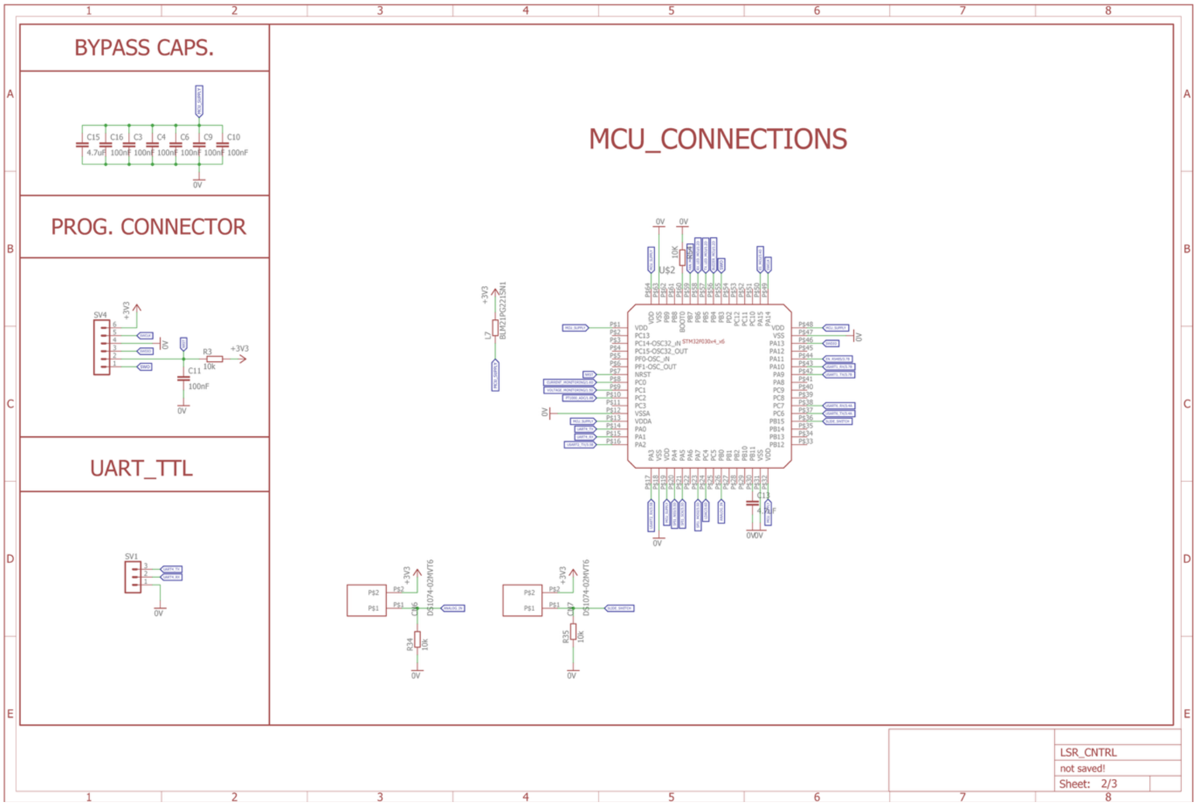

b) Electronic control unit schematics sheet2

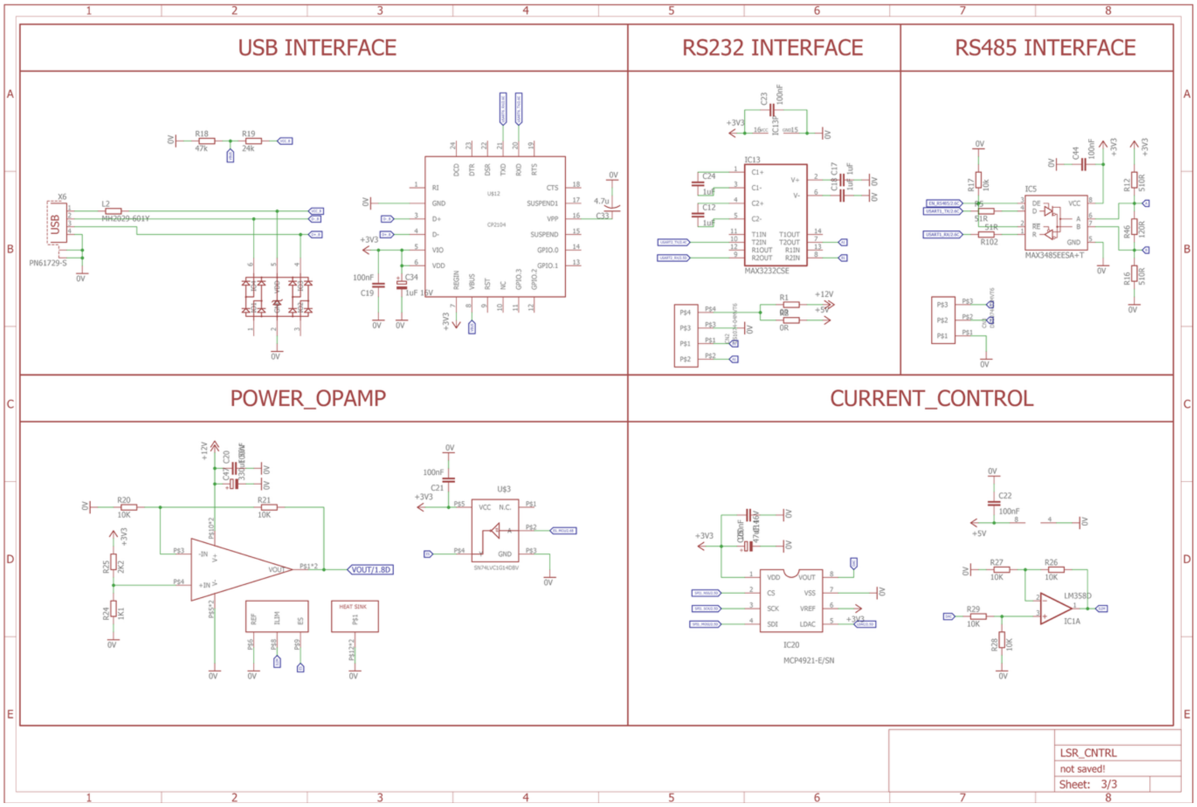

c) Electronic control unit schematics sheet3

Supplementary Figure 3: Electronic control unit simplified schematics

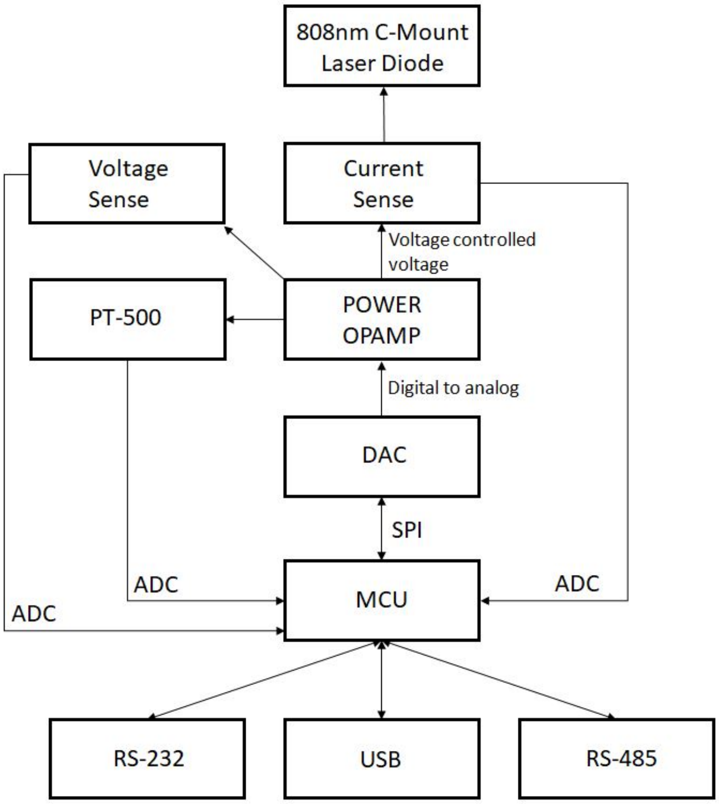

Supplementary Figure 3: The electronic control unit is primarily responsible for the operation of the system. The system simultaneously controls the output power of the laser diode while measuring both the current and voltage applied to the diode. Additionally, the output voltage is regulated through the power operational amplifier, which is connected to the digital-to-analog converter (DAC). The DAC is controlled by the microcontroller unit (MCU) via SPI. The controlling process generates heat on the power operational amplifier (op-amp). Therefore, an aluminum heat sink is attached to the op-amp, and the temperature of the op-amp is also measured using a PT-500 sensor. Throughout the measuring process, the heat, current, and voltage are connected to the analog-to-digital converter (ADC) of the MCU. The USB layer is connected to PC and RS-232 layer is connected to IR array unit. RS-485 communication layer of the system is reserved for future use. When the USB is connected to a computer, a virtual COM port automatically opens in the operating system. So that any computer with a free serial terminal software can use the system.

Supplementary Figure 4: Temperature unit PCB design.

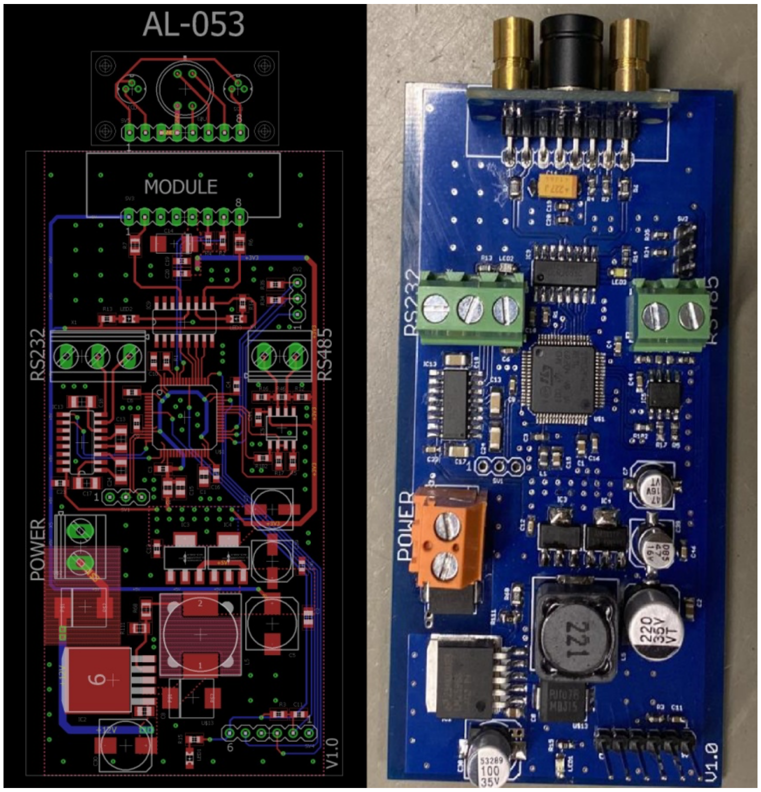

Supplementary Figure 4: A two-layer PCB design was preferred as a cost-effective solution. The PCB material selected was FR4 for its physical durability, while aluminum was chosen for the enclosure material to enhance durability further. AL-053 code written on top of the Supplementary Figure 2 shows the manufacturer product number of the enclosure. Connectors X1 and X4 serve as communication interfaces. Additionally, SV3 is designated for the IR module daughterboard. The connector X5 is the power input of the device and the input voltage of the device is 12V. Connector SV4 is a male 2.54mm pin header for debugging and programming the MCU.

The PCB properties:

- Material: FR-4 TG150
- Layer: 2 Layers
- Finished copper: 35um
- Thickness: 1.6mm
- Surface finish: HASL with lead
- Size: 50.8 x 99.8 mm

Supplementary Figure 5: IR unit schematic

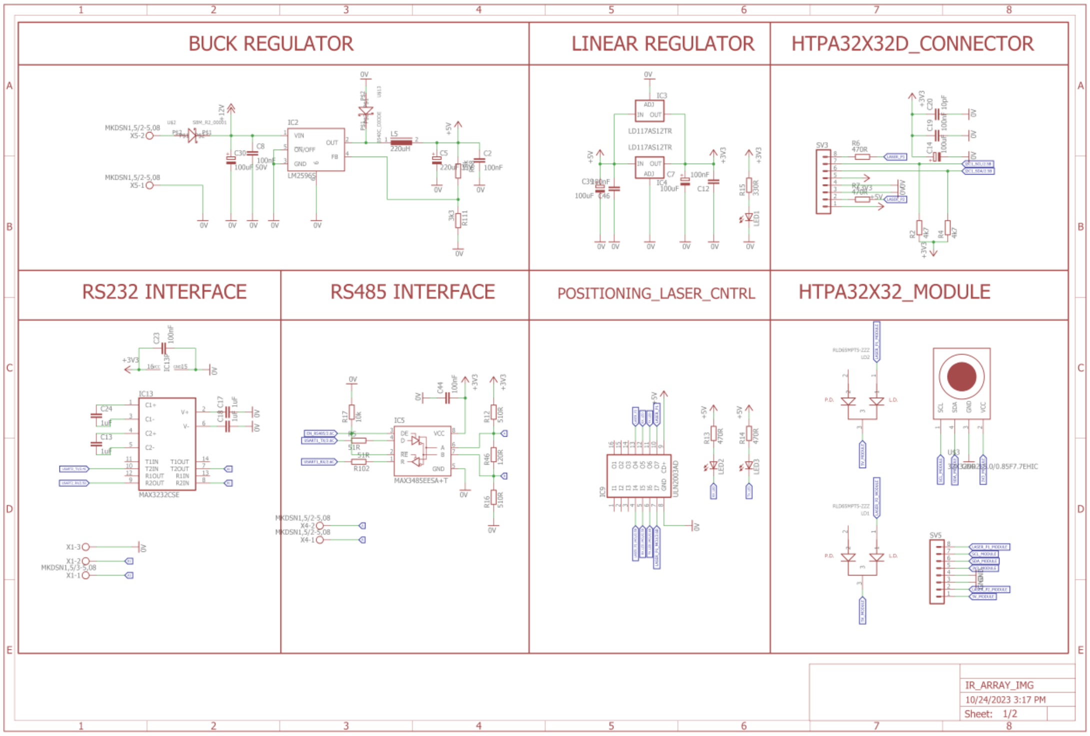

a) IR unit schematics sheet1

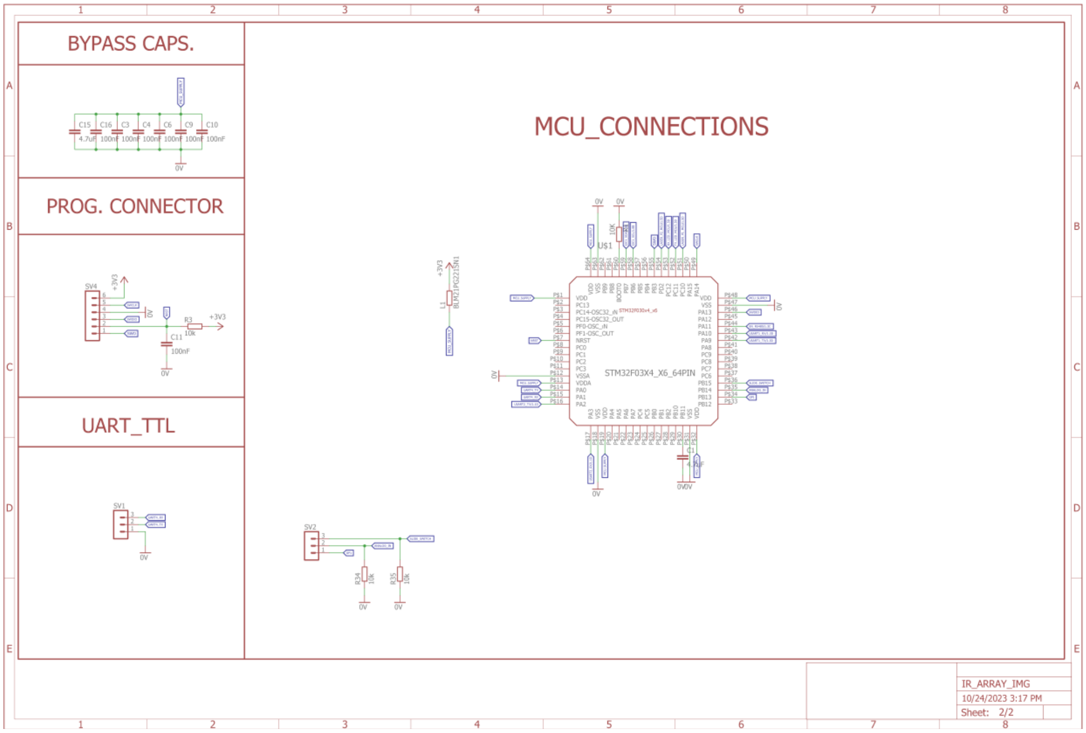

b) IR unit schematics sheet2

Supplementary Figure 6: IR unit simplified schematics

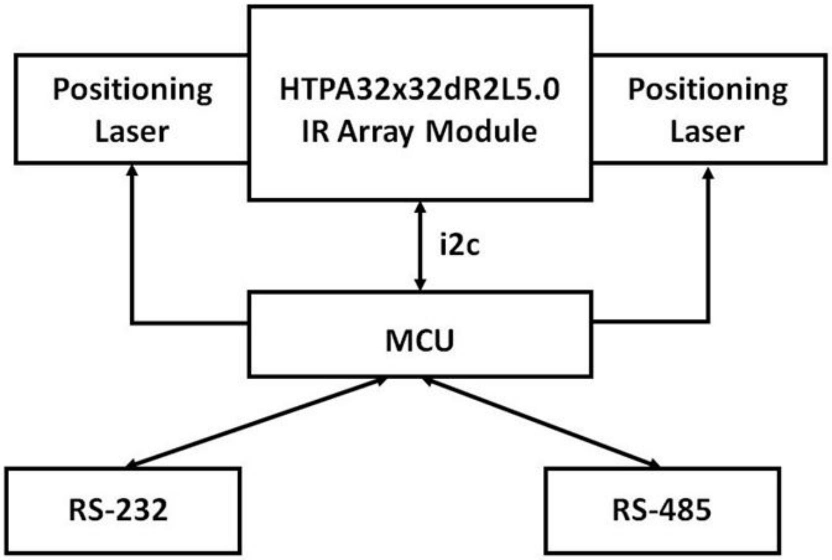

Supplementary Figure 6: IR array unit consists of 4 main sections as seen in Supplementary Figure 6. These are positioning lasers, IR Array module, MCU, and communication interfaces, respectively. The IR array module communicates with MCU via I2C, and the temperature values of 32x32 pixels measured at 330ms intervals are transmitted to the MCU. The instantaneous surface temperature information is obtained, and the average of the temperature values of 4 pixels located at 15th row-15th column, 16th row-15th column, 15th row-16th column, and 16th row-16th column in the center is calculated. This average temperature information is then sent to the ECU via RS-232 at a speed of 115200bps. On the other hand, the RS-485 communication interface is connected to the PC. The temperature information of 32x32 pixels is responded at a speed of 115200bps upon the command received from the PC, and it is displayed on the interface program. The positioning lasers are 5mW 650nm dot lasers as seen in Supplementary Figure 2 and placed at the both sides of IR array module. The main purpose of these lasers is to facilitate the usage of the IR array unit during experiments. The positioning lasers toggle in every 1s and the pointed area is the location where IR array unit measures. RS-232 serial communication, RS-485 circuit and power layers are identical with the electronic control unit as seen in IR unit schematics. Moreover, the MCU used in the IR array unit, with a core speed of 180 MHz and connectivity capabilities, is identical to the one used in the ECU.

Supplementary Figure 7:

a) Laser Irradiation Control Algorithm

```
void Laser_Target_Temp_Check()
{
	extern float gTemp;
	extern volatile uint8_t gCoefficient;
	extern uint8_t tPer;

	if(laser_time.timeout == 0)
	{
		return;
	}

	float temp_difference = (float)((gTemp - laser_time.target_temp));
	// Calculate proportional power adjustment
	percentage = (int)((50+gCoefficient)  - temp_difference * (50-gCoefficient));
	tPer=percentage;
	// Ensure the percentage is within the valid range [0, 100]
	percentage = (percentage > 100) ? 100 : percentage;
	percentage = (percentage < 20) ? 20 : percentage;

	IO_control_laser(1, 0xFFFFFFFF, percentage);

}
```

b) Temperature Increase Rate Detection Function

```
void HAL_TIM_PeriodElapsedCallback(TIM_HandleTypeDef *htim)
{
  if(htim == &htim6)
  {
	HAL_GPIO_WritePin(RX_LED_GPIO_Port, RX_LED_Pin, GPIO_PIN_RESET);

	if(Mode_Flag==1)
      {
		gSlope[iCounter]=gTemp;

		iCounter++;

		if(iCounter>250)
		{
	       if((gSlope[249]-gSlope[0])>=5)
	       {
		gCoefficient=0;
	       }
	       else
	       {
		gCoefficient=30;
	       }
			Mode_Flag=0;
		    iCounter=0;
	       } } } }
```

Supplementary Figure 8:

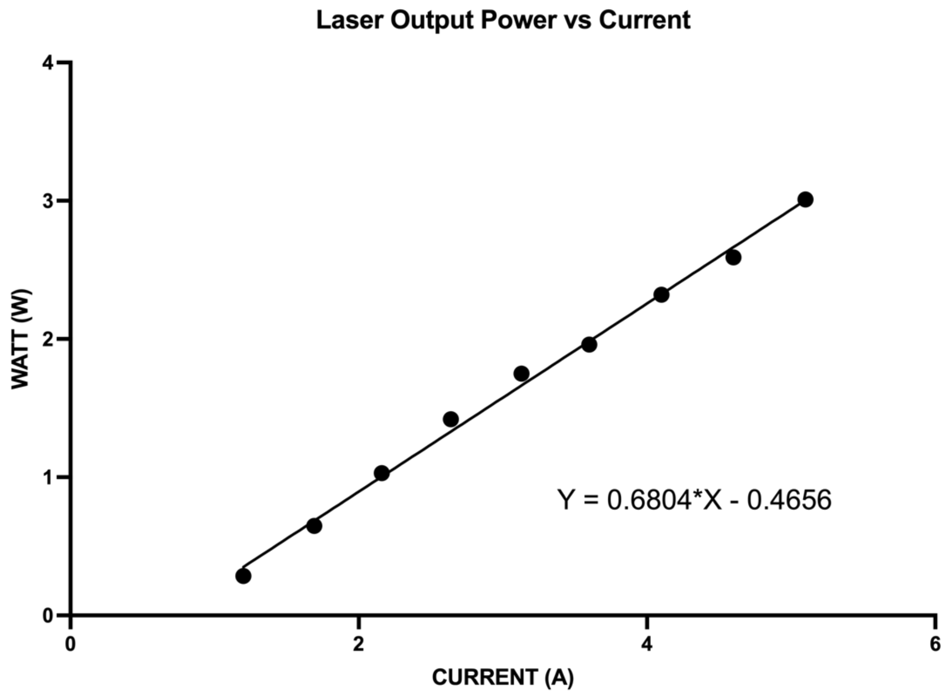

Supplementary Figure 8: Curve and formula used for the conversion between current and laser output power. The curve fitting was performed using GraphPad Prism 10.
